# Machine Learning Clinical Decision Support for Interdisciplinary Multimodal Chronic Musculoskeletal Pain Treatment: Prospective Pilot Study of Patient Assessment and Prognostic Profile Validation

**DOI:** 10.2196/65890

**Published:** 2025-05-09

**Authors:** Fredrick Zmudzki, Rob J E M Smeets, Jan S Groenewegen, Erik van der Graaff

**Affiliations:** 1 Department of Rehabilitation Medicine, Care and Public Health Research Institute, Faculty of Health, Life Sciences and Medicine, Maastricht University, Universiteitssingel 40, Room 3.544, P.O. Box 616, 6200 MD, Maastricht, 6229 ER, The Netherlands, 31 433882160; 2 Epoque Consulting, Sydney, Australia; 3 Social Policy Research Centre, University of New South Wales, Sydney, Australia; 4 CIR (Expertisecentrum chronische pijn), Eindhoven, The Netherlands; 5 Pain in Motion International Research Group, Brussels, Belgium; 6 CIR (Expertisecentrum chronische pijn), Rotterdam, The Netherlands; 7 CIR (Expertisecentrum chronische pijn), Amsterdam, The Netherlands

**Keywords:** chronic pain, musculoskeletal pain, machine learning, interdisciplinary care, clinical decision support, musculoskeletal, prognostic

## Abstract

**Background:**

Chronic musculoskeletal pain (CMP) impacts around 20% of people globally, resulting in patients living with pain, fatigue, restricted social and employment capacity, and reduced quality of life. Interdisciplinary multimodal pain treatment (IMPT) programs have been shown to provide positive and sustained outcomes where all other interventions have failed. IMPT programs combined with multidimensional machine learning predictive patient profiles aim to improve clinical decision support and personalized patient assessments, potentially leading to better treatment outcomes.

**Objective:**

We aimed to investigate integrating machine learning with IMPT programs and its potential contribution to clinical decision support and treatment outcomes for patients with CMP.

**Methods:**

This prospective pilot study used a machine learning prognostic patient profile of 7 outcome measures across 4 clinically relevant domains, including activity or disability, pain, fatigue, and quality of life. Prognostic profiles were created for new IMPT patients in the Netherlands in November 2023 (N=17). New summary indicators were developed, including defined categories for positive, negative, and mixed prognostic profiles; an accuracy indicator with high, medium, and low levels based on weighted true- or false-positive values; and an indicator for consistently positive or negative outcomes. The consolidated reporting guidelines checklist for prognostic machine learning modeling studies was completed to provide transparency of data quality, model development methodology, and validation.

**Results:**

The machine learning IMPT prognostic patient profiles demonstrated high accuracy and consistency in predicting patient outcomes. The profile, combined with extended new prognostic summary indicators, provided improved identification of patients with predicted positive, negative, and mixed outcomes, supporting more comprehensive assessment. Overall, 82.4% (14/17) of prognostic patient profiles were consistent with clinician assessments. Notably, clinician case notes indicated the stratified prognostic profiles were directly discussed with around half (8/17, 47.1%) of patients. Clinicians found the prognostic patient profiles helpful in 88.2% (15/17) of initial IMPT assessments to support shared clinician and patient decision-making and discussion of individualized treatment planning.

**Conclusions:**

Machine learning prognostic patient profiles showed promising contributions for IMPT clinical decision support and improving treatment outcomes for patients with CMP. Further research is needed to validate these findings in larger, more diverse populations.

## Introduction

Chronic musculoskeletal pain (CMP) is a prevalent and debilitating condition that significantly impacts the quality of life for millions of individuals worldwide [1]. Patients with CMP often experience long-term pain, where all available treatments have been ineffective, resulting in debilitating pain; fatigue; limited mobility; and associated reduced social activity, employment, and quality of life. Interdisciplinary multimodal pain treatment (IMPT) has shown positive outcomes for CMP, with over 50% of patients reporting positive and sustained improvement [2-8]. CIR (Expertisecentrum chronische pijn), a multicenter pain rehabilitation clinic in the Netherlands, provides a 10-week IMPT program for patients with CMP, reporting consistent positive outcomes [8,9]. The IMPT program supports patients with CMP in modifying their behavior and assisting with pain management, focusing patient attention on specific value-based goals rather than fighting pain.

Recent advancements in machine learning have opened new avenues for enhancing clinical decision support and personalizing IMPT patient assessments. The promising potential of machine learning models to assist clinical decision support for IMPT patients has been previously reported [10-12]. Specifically, machine learning methods can assist with assessing multiple predictive outcomes to provide a stratified prognostic patient profile. This multidimensional approach is essential because, given the complexity of evaluating CMP, no single outcome measure is sufficiently reliable. Clinicians consider various dimensions when assessing patients for IMPT. Machine learning prognostic profiles have demonstrated potential in enhancing clinician assessments for IMPT and identifying individual patient objectives.

The primary objectives of this study were to (1) assess the effectiveness of machine learning predictive patient profiles in improving clinical decision support for IMPT programs; (2) evaluate the accuracy and consistency of prognostic assessments derived from multidimensional machine learning models; and (3) develop new summary indicators, including redefined prognostic categories and accuracy guides, to better assist clinicians and patients in interpreting the prognostic profiles and making informed treatment decisions.

Implementing machine learning prognostic profiles requires clinician collaboration and clear demonstration of their capabilities. Recent research has shown potential pathways to validate and build clinicians’ confidence in using machine learning for IMPT decision support [13,14]. This pilot study aimed to investigate integrating machine learning with IMPT programs and its potential contribution to clinical decision support and treatment outcomes for patients with CMP.

## Methods

### Study Design

This pilot study evaluated integrating machine learning predictive patient profiles with IMPT programs. Data included all patients with CMP referred for CIR program assessments in Eindhoven over 3 weeks in November 2023 (N=18). Eligible participants were adults 18 or older with CMP and at least 6 months of pain history, excluding those with severe psychiatric disorders, active substance abuse, or ongoing pain-related litigation [8,9]. One pilot study patient had experienced severe traumatic events and was clinician-assessed for referral to seek proper care in mental health and was not suitable for IMPT. For this patient, the IMPT prognostic profile was not relevant, as the IMPT outcome framework questionnaires do not include instruments to assess trauma. Due to the incorrect referral, this patient was excluded from the study, resulting in the final IMPT patient pilot study group (N=17).

**Figure 1.**
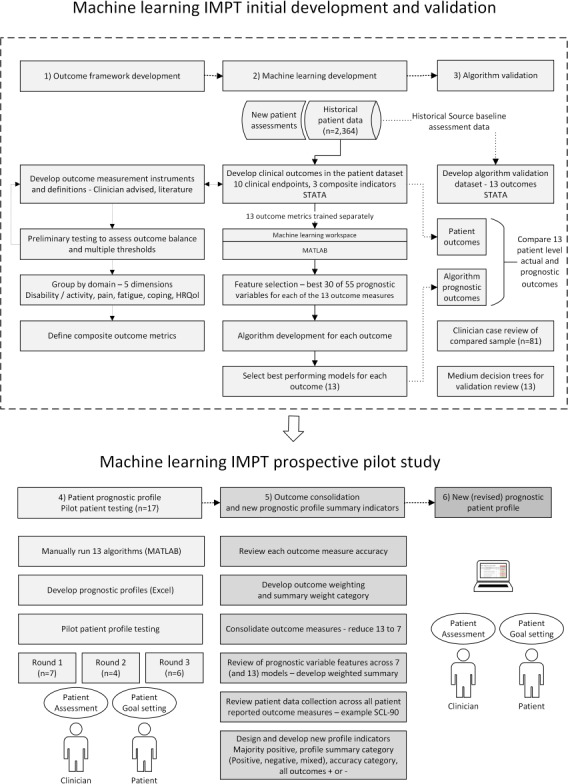
Diagram of machine learning IMPT pilot study process. Algorithm development was previously undertaken for multiple classification models, selecting the best performing model for each of the final 7 outcome measures. Dashed section indicates previously reported IMPT model development [10]. HRQoL: health-related quality of life; IMPT: interdisciplinary multimodal pain treatment; SCL-90: Symptom Checklist-90.

The pilot study used multidimensional machine learning models that were previously developed to predict patient outcomes based on a comprehensive set of clinical, demographic, and psychosocial variables derived from historical data of previous IMPT patients (N=2364) [10]. The present pilot testing study applied these machine learning algorithms to generate prognostic profiles for new IMPT patients prior to their initial assessment. As illustrated in Figure 1, over the course of 3 weeks, each profile was prepared manually, involving finalization of patient data and multiple processing steps.

### Data Sources and Preprocessing

Data collected from IMPT-referred patients in preparation for baseline assessments included demographics, clinical conditions, patient-reported outcomes, and a comprehensive evaluation of pain intensity, physical function, emotional well-being, social participation, and other relevant variables. The baseline data were used with previously trained algorithms to generate prognostic profiles for new patients using CIR data from the Asterisque system (electronic patient file). Clinicians assess some prognostic data items during initial patient examinations, such as indication for eye movement desensitization and reprocessing registration and pain diagnosis (level of complexity known as WPN classification). Other assessments, like the 6-minute walking test and 5 times sit-stand trials (performed at the actual start of the IMPT), were imputed based on age and gender averages from IMPT patient data, with supplementary checks to confirm consistency in prognostic profiles.

A machine learning development and validation checklist was used to ensure the quality and consistency of the models [15]. The checklist included steps for data preprocessing, model training, validation, and evaluation; details are provided in Multimedia Appendix 1.

### Ethical Considerations

All records in the patient dataset were deidentified with a unique patient number, and clinician patient assessments of positive or negative IMPT referrals and case notes were reported for validation of each patient prognostic profile. The Medical Research Ethics Committee Isala Zwolle, the Netherlands, previously reviewed the initial study (case number assigned: 200510), and as all patients have consented to the use of their data for research, further ethics approval was not required. This satisfies that the study was conducted in accordance with the principles outlined in the Declaration of Helsinki.

### Revised and New Prognostic Profile Summary Indicators

The primary pilot validation outcome measure was the accuracy and consistency of prognostic assessments derived from the machine learning models in support of clinician and IMPT team-assessed prognosis. Secondary outcome measures included the effectiveness of the predictive patient profiles in improving clinical decision support, the development of new summary indicators, and the value of these indicators for clinician and patient shared decision-making. In this context, in line with the complexity of IMPT treatment and clinician assessment, a review of the multidimensional outcome framework was undertaken to consider how the results across the prognostic profile could best be summarized to assist clinician and patient interpretation.

Five new summary indicators were established (Table 1). The initial indicator shows positive or negative based on a majority count of positive outcomes (indicator 1), as well as the actual number of positive outcomes (indicator 2). A new prognostic profile summary indicator (indicator 3) was created to more clearly identify positive or negative profiles. The negative profile is defined as all 7 outcomes being consistently negative (zero positive), while the positive profile requires 3 or more positive outcomes. Profiles that do not fit either category are labeled as “mixed” and need further assessment. These summary categories supplement the complete set of stratified prognostic outcomes that are individually provided for clinician evaluation and optional patient discussion.

Additionally, each prognostic model was evaluated to create a weighted summary indicator of overall accuracy (summary indicator 4). This accuracy category was developed by summing true-positive rates (TPRs) and true-negative rates (TNRs) across each machine learning model for each outcome. This figure provides a weighted proxy indicator to adjust for outcome models with high TPRs or TNRs, derived by summing the opposite TPR or TNR for each predicted positive or negative outcome. For example, an outcome with an estimated TPR of 0.98 added the related TNR of 0.02 to the weighted accuracy score, reflecting a low contribution to the total profile. The resulting true-positive and true-negative weighted scores were then grouped into a prognostic profile accuracy category defined as low (<3), medium (≥3 and <4), and high (≥4). A supplementary indicator (indicator 5) was also developed to emphasize profiles where all 7 indicators were consistently positive or negative. While the full profile shows the number of positive or negative outcomes, this indicator clearly highlights these significant cases compared to mixed outcomes.

**Table 1.** Revised and new interdisciplinary multimodal pain treatment (IMPT) prognostic patient profile summary indicators. Source: Machine learning IMPT prognostic patient profile pilot study (n=17).

IMPT prognostic patient profile indicator	Positive	Other	Negative
1. Positive majority (positive outcomes)	≥4 of 7	N/A^a^	<4 of 7
2. Number of positive outcomes (outcome count)	4, 5, 6, or 7	N/A	1, 2, or 3
3. Prognostic profile summary (positive outcomes)	≥3 of 7	Mixed=1 or 2	0
4. Profile accuracy category (weighted TPR^b^ and TNR^c^)	High	Medium	Low
5. All outcomes, positive or negative	All of 7	N/A	All of 7

a N/A: not applicable.

b TPR: true-positive rate.

c TNR: true-negative rate.

### Statistical Analysis

Statistical analyses compared the accuracy and consistency of the IMPT machine learning prognostic profiles with clinician assessments in the context of IMPT clinical decision support; that is, how consistent are the machine learning profiles with clinician prognoses to start IMPT. Descriptive statistics summarized baseline characteristics and outcomes; categorical variables are presented as numbers and percentages, and continuous variables as mean and SD. Fisher exact test was used to measure associations due to the small pilot study sample (N=17) versus initial models (N=2364). Details on feature selection and individual machine learning model IMPT outcomes have been previously reported [10].

Data analysis and preprocessing were completed with Microsoft Excel 365 and STATA (version 16.1; StataCorp LP). Algorithm prediction was undertaken individually for each of the 7 outcome models using the MATLAB Statistics and Machine Learning Toolbox (Release 2023b). Prognostic patient profile results were transposed back to Microsoft Excel for clinician and patient review.

## Results

In total, 17 patients with CMP participated in the study. The mean age of the participants was 45.3 (SD 12.4) years, with 10 females and 7 males. Most participants had a history of pain lasting more than 5 years. Baseline characteristics are detailed in Multimedia Appendix 1. As all patients have been referred for IMPT, patients in the pilot study group were diagnosed with CMP and classified through the Working Group on Pain Rehabilitation in the Netherlands (Werkgroep Pijnrevalidatie Nederland (WPN)). Based on this rating of the complexity of pain symptoms, the majority (64.7%) were assessed as WPN 3 chronic pain syndrome and 35.3% as WPN 4 (maximal score), consistently showing a clear indication of psychosocial factors underlying chronic pain and disability. Most participants lived with a partner, were employed, and used pain medication.

**Figure 2.**
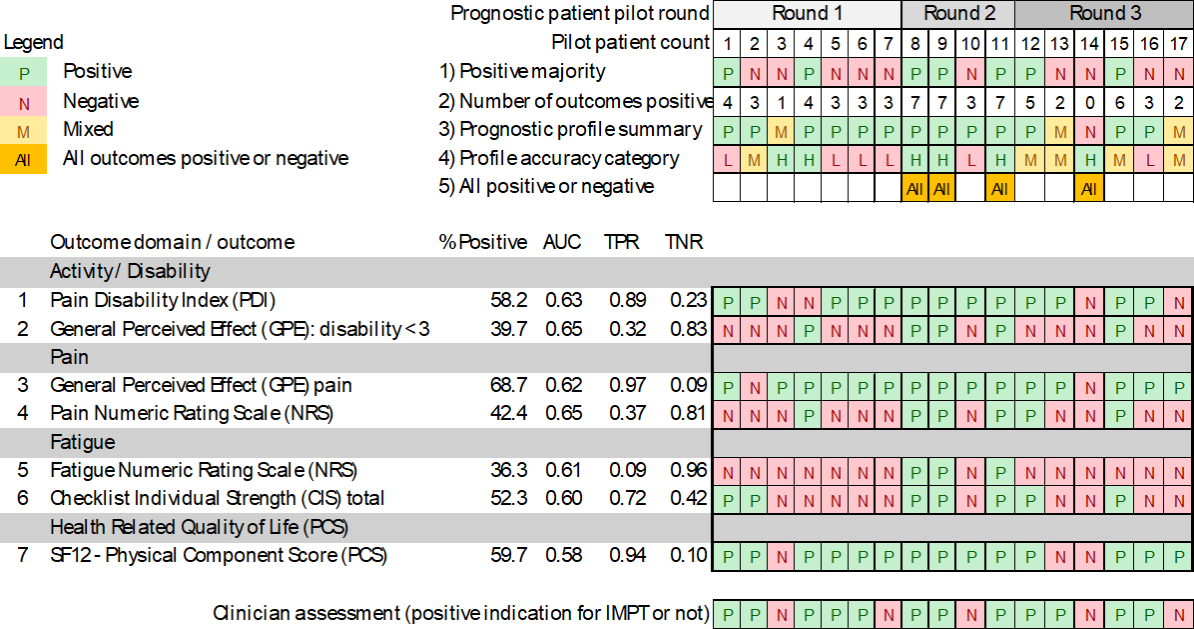
Pilot study prognostic patient profiles with 7 outcome measures and new summary indicators. Source: Machine learning interdisciplinary multimodal pain treatment (IMPT) prognostic patient profile pilot study (N=17). Profile accuracy: H=high, M=medium, L=low. AUC: area under the curve; M: mixed; N: negative; P: positive; TPR: true-positive rate; TNR: true-negative rate.

The machine learning IMPT prognostic patient profile with new summary indicators demonstrated high accuracy and consistency with clinician assessment in 82.4% (14/17) of cases (Figure 2). In 2 of the 3 mixed cases, the profiles were predominantly negative (patients 3 and 17 in Figure 2), and the clinician confirmed them as negative during assessment. The remaining mixed profile (patient 13) included 2 positive prognostic outcomes and was clinician-confirmed as positive. The prognostic profile was not used in 2 cases, one where the patient turned out to have no request for help with pain rehabilitation and another where the planned assessment was rescheduled beyond the pilot study period.

Notably, clinician assessment indicated the stratified prognostic profiles were directly discussed with around half (8/17, 47.1%) of patients and were reported as helpful for IMPT discussion, shared decision-making, and individualized planning. The patient review discussion was valuable for confirming profiles that were mostly positive, verifying the positive decision. The review discussions were also reported as helpful in 2 additional mixed profile cases, one where review of predicted negative outcomes helped the patient decide not to proceed with IMPT, even though one summary indicator was positive (patient 3), and another with mostly negative outcomes, which also helped confirm a decision not to proceed with IMPT (patient 17).

Collectively the prognostic patient profile indicators were clearly consistent with positive and the single negative clinician assessment in 12 cases and indirectly consistent through 2 mixed profiles (14/17), as above. A further example of machine learning prognostic profile value was a predicted positive profile (patient 10), even though the joint clinician and patient decision was not to proceed with treatment. In this case the profile showed the minimum of 3 positive outcomes out of 7, and the profile accuracy category was low. Combining this case with the previous 14 clinician-consistent results showed the prognostic patient profiles were helpful in 88.2% (15/17) of initial IMPT assessments. Clinician experience during the study indicated that the predictive profiles provided valuable insights into patient outcomes and facilitated more informed decision-making regarding individualized treatment plans.

The new prognostic profile summary indicators included a profile accuracy category (indicator 4). Although the pilot study is a small sample, the results consistently indicated that all 6 patients with a “high” accuracy category were confirmed to have a clinician-consistent prognostic profile. Many of this high-accuracy category group (4/6) had a clear positive or negative profile summary, highlighted by the final indicator where all 7 outcomes are consistently positive or negative.

**Figure 3.**
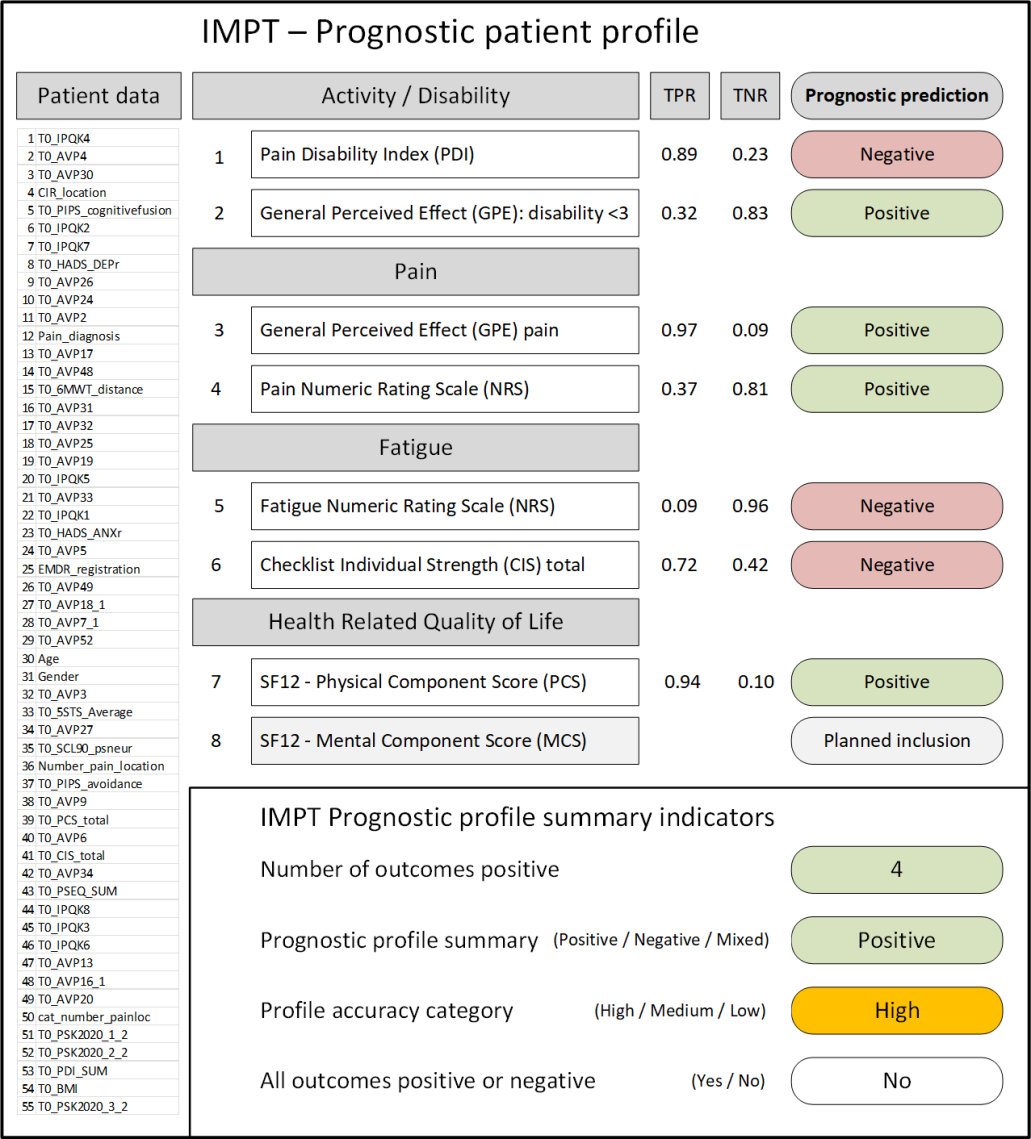
IMPT prognostic patient profile and summary indicators—pilot study example. Example patient number 4 prognostic profile with actual pilot testing study data results. Patient data section is shown for illustrative purposes, representing the 55 baseline prognostic variables and values that would appear on the patient profile. CIS: Checklist Individual Strength; GPE: Global Perceived Effect (disability); IMPT: interdisciplinary multimodal pain treatment; NRS: Numeric Rating Scale; PDI: Pain Disability Index; SF-12 MCS: Short-Form Quality of Life Survey Mental Component Score; SF-12 PCS: Short-Form Quality of Life Survey Physical Component Score; TNR: true-negative rate; TPR: true-positive rate.

The above summary (Figure 2) presents results for all pilot study patients to show performance and overall results. However, the individual prognostic patient profile as used in IMPT clinical assessment provides clearly presented summary results for each patient. The IMPT prognostic patient profile included all patient baseline and demographic data for reference, the stratified outcomes by outcome dimension, and the prognostic prediction clearly displayed for each outcome. The example in Figure 3 shows indicative profile content, which would potentially be further designed and customized into a patient prognostic profile application. This illustration indicates the planned inclusion of an additional outcome measure for the Short-Form Quality of Life Survey (SF-12) Mental Component Score (MCS), as IMPT data for this scale is now available. The bottom section presents the prognostic profile summary indicators, in this case showing a positive summary with a high level of accuracy confidence.

## Discussion

### Overview

Chronic pain is a complex condition that needs assessment of multiple patient outcomes to create personalized treatments. Managing chronic pain is complicated due to its subjective nature and the variability in patient responses to treatments. In this context, the pilot study investigated the performance of a novel machine learning prognostic patient profile, which combined 7 separately trained outcome models across 4 clinically meaningful domains into a single prognostic patient profile [10]. The study investigated how this multidimensional approach assisted clinical decision support, shared clinician and patient decision-making, and individualized treatment planning.

### IMPT Improved Program Outcomes and Efficiency

The pilot study externally validated the IMPT predictive patient profile, showing its potential to enhance chronic pain treatment and cost-effectiveness. By introducing new summary indicators and weighted accuracy measures, the study improved the tool’s precision in predicting patient outcomes. It also identified key variables for better data utilization, suggesting that some, like the Symptom Checklist-90 (SCL-90) questionnaire, could be removed to reduce patient burden. The machine learning feature selection process can help minimize redundant information and optimize high-impact variables, leading to more effective chronic pain management. The IMPT prognostic variables weighted by minimum redundancy maximum relevance scores are provided in Multimedia Appendix 1.

Additionally, the overlap with current IMPT patient systems indicates potential for optimizing data usage. The pilot study revealed that clinicians spend approximately 15 minutes per patient collecting initial baseline data. Having the comprehensive prognostic profile available could save this time for more complex assessments. Separately, the prognostic patient profile might play a more nuanced role in patient assessment. These profiles could help identify a subgroup of patients who might benefit from an initial 4-week treatment period to further evaluate IMPT potential before proceeding to the full 10-week program. This approach could contribute to better assessment pathways and improved patient outcomes.

Furthermore, in a longer term perspective, the societal value of IMPT is linked to the positive patient pathways that emerge following participation in the program, which may persist for many years or even decades. Preliminary research indicates that the CIR program is likely to be cost-effective, with long-term results demonstrating significantly enhanced self-rated work capacity [2,8]. This offers a promising outlook for further efficiency gains and improved patient outcomes, where machine learning-driven predictive patient profiles may assist clinicians and patients in assessment and program engagement. Long-term outcomes could facilitate a return to working life, reduce the need for formal and informal care, and enhance quality of life.

### Artificial Intelligence Advances in Chronic Pain Health Care

The use of artificial intelligence in health care, including chronic pain diagnosis and treatment, has become increasingly prominent [11,16-18]. However, most machine learning research has investigated pain diagnosis and progression to chronic pain, which had already been completed for the pilot study group, which consisted of patients with long-term CMP. All patients had previous consultations with at least 1, and up to 20, care providers for diagnostics and treatment, in addition to visiting a physical therapist in primary care. Patient pain complexity was reflected in WPN levels 3 or 4, indicating the presence of multiple biopsychosocial factors contributing to persistent pain, associated disability, and reduced quality of life. This suggested that monodisciplinary treatment would have been insufficient. This pilot study, however, went beyond diagnosing chronic pain and used machine learning to predict the success of IMPT for patients with CMP, especially when other treatments had failed.

### Single Versus Multidimensional Outcomes

Many machine learning studies focus on predicting single outcomes for chronic pain treatment, such as whether a patient’s pain will improve with specific therapy. These studies primarily address binary clinical decisions rather than developing multioutcome patient profiles. For example, one study used machine learning with features from dynamic surface electromyography readings to predict which patients with low back pain would respond to rehabilitation [19], showing high accuracy, possibly due to a specific signal in the data. Another study evaluated if patients with low back pain should seek professional care or self-manage, achieving around 70% accuracy [20].

A further single-outcome approach developed a neural network model to predict which patients with chronic pain and foraminal spinal stenosis would benefit from a transforaminal epidural steroid injection [11]. These single-outcome approaches focus on clinical procedures rather than personalized multimodal treatments. Overall, while high-resolution imaging may provide precise diagnoses, it cannot be compared directly to complex patient-reported chronic pain data.

### Supervised and Unsupervised Learning

In addition to supervised machine learning investigated in the pilot study, unsupervised methods have been used to identify patient subgroups for better pain treatment selection [21]. For example, clustering has been applied to patients with fibromyalgia to find distinct subgroups based on symptom profiles like pain intensity, anxiety, and sleep quality [22]. Clustering helps understand mechanisms or prognoses for varied treatments. It aids in patient stratification and decision support but does not give direct recommendations. Clinicians can use clusters to guide treatment by matching new patients to typical outcomes. Clustering differs from supervised approaches as it explores and identifies patterns without assumptions. Furthermore, clusters require clinical interpretation and validation and do not have accuracy metrics like those found in predictive models. Recent research has also explored the mix of supervised and unsupervised methods to investigate underlying subgroups of patients with chronic pain [23]. Given the characteristic heterogeneity of patients with CMP, this research may help better identify prognostic subgroups, which could provide improved direction for ongoing supervised and reinforced IMPT machine learning development.

### Prognostic Profile Implementation

An implicit machine learning research direction highlights the importance of carefully planned implementation to build confidence in these new methods and achieve acceptance from clinicians and patients. The challenges to successful implementation included training data quality, complexity of pain physiology, comprehensive validation of predictive models, privacy, biases, and ethical considerations [10-12,24]. Proactively anticipating and addressing each of these barriers from the outset is essential for the successful integration of machine learning into IMPT clinical decision support systems.

The machine learning pilot study involved continuous clinician collaboration in line with research indicating that clinician involvement during the design and validation phases is crucial for supporting potential implementation acceptance [25]. To further support design consistency and transparency, the pilot study built on a previously completed clinician checklist for algorithm assessment, which considered aspects like data quality, validation, and ethical concerns [10,26]. This checklist has now been extended with the Consolidated Reporting Guidelines for Prognostic and Diagnostic Machine Learning Modeling Studies, further enhancing standardization and building confidence for clinical integration [15].

### External Validation

The pilot study has demonstrated initial external validation, as the prognostic profiles have been prospectively investigated with new patients who were not included in algorithm training data. A further interesting aspect has been the consistency with conventional logistic regression models as a form of cross-validation for the machine learning prognostic profiles. Separate research into IMPT logistic regression prediction models has investigated 3 of the 7 outcome dimensions used in the machine learning profiles and found consistent results for corresponding outcomes [27]. The logistic regression study examined the Pain Disability Index (outcome 1 in Figure 2), the General Perceived Effect (outcome 2), and the SF-12 Physical Component Score (outcome 7). These results reinforce the reliability and validity of the machine learning methods, and this cross-validation strengthens confidence in the prognostic tool’s ability to provide accurate and useful predictions for IMPT patient outcomes. Importantly, the logistic regression results indicate that the SF-12 MCS was the highest performing model, and is a second outcome measure in the health-related quality of life dimension. This outcome was not trained during initial IMPT machine learning development as the data were not yet available. However, this measure is currently being integrated into the machine learning prognostic patient profile (shown as outcome 8 in Figure 2), suggesting further potential positive contribution beyond the pilot study results.

### Limitations

Although the results are promising, this study has some limitations. As the IMPT patient data extraction and preparation were time-consuming and the machine learning models involved substantial manual effort to individually run across each outcome and collate into each patient profile, it was not feasible to run the pilot study for an extended period. This limited the pilot study group to a small sample of 17 patients over the 3-week project. For this reason, the results are presented as preliminary and have focused on individual profile analysis and clinician validation, rather than statistical significance.

Additionally, all pilot study patients were assessed at a single CIR location in Eindhoven, limiting the scope to examine other patient cohorts, clinicians, and potentially other IMPT program characteristics. The results are provided as indicative and descriptive, and the small sample and single-center study may affect the generalizability of the findings. Future research should aim to confirm these findings in larger, more diverse IMPT populations and investigate the long-term contribution of machine learning to clinical decision support and patient outcomes.

### Conclusions

Integrating machine learning with IMPT programs can enhance clinical decision support and potentially improve treatment outcomes for patients with CMP. Machine learning predictive patient profiles can complement clinician assessments by providing insights into specific patient outcomes and facilitating informed clinician and patient shared decision-making. New summary indicators can further enhance predictive profile interpretation for consideration of whether IMPT is likely to provide a positive treatment pathway. Continued research and development in this area are necessary to fully realize the potential of machine learning in clinical practice and to ensure its successful implementation and acceptance by clinicians and patients.

## Supplementary material

10.2196/65890 Multimedia Appendix 1 Machine learning development and validation checklist, participants’ baseline characteristics, and data on prognostic patient profile.

## Abbreviations

CIR: Expertisecentrum chronische pijn
CMP: chronic musculoskeletal pain
IMPT: interdisciplinary multimodal pain treatment
MCS: Mental Component Score
SCL-90: Symptom Checklist-90
SF-12: Short-Form Quality of Life Survey
TNR: true-negative rate
TPR: true-positive rate
WPN: Werkgroep Pijnrevalidatie Nederland
