# Supplementary material for: Machine Learning Clinical Decision Support for Interdisciplinary Multimodal Chronic Musculoskeletal Pain Treatment: Prospective Pilot Study of Patient Assessment and Prognostic Profile Validation
Source: JMIR Rehabil Assist Technol. 2025 May 9;12:e65890. doi: 10.2196/65890 (PMC12083736; doi:10.2196/65890)

**Multimedia Appendix 1**

## Consolidated reporting guidelines for prognostic and diagnostic machine learning modelling studies

# Author Checklist

This reporting checklist indicates where each item is documented in our IMPT prognostic patient profile study, or comments where a response is N or NA. These consolidated reporting guidelines for prognostic machine learning modeling studies have been independently developed and reported (Klement & El Emam, 2023). As this project is a follow up external validation phase many details of machine learning model development have been reported in our previous publication and are noted where relevant.

| **#** | **Item** | **Y** | **N** | **NA** | **Location / Reasoning** |
| --- | --- | --- | --- | --- | --- |
| **Study Details** | | | | | |
| 1.1 | *The medical/clinical task of interest* | ✓ |  |  | 1. Introduction |
| 1.2 | *The research question* | ✓ |  |  | 1. Introduction |
| 1.3 | *Current medical/clinical practice* | ✓ |  |  | 1. Introduction and initial machine learning prognostic patient profile development (Zmudzki & Smeets, 2023) |
| 1.4 | *The known predictors and confounders to what is being predicted / diagnosed* | ✓ |  |  | Machine learning prognostic patient profile outcome framework (Zmudzki & Smeets, 2023) |
| 1.5 | *The overall study design* | ✓ |  |  | 2. Methods |
| 1.6 | *The medical institutional setting(s)* | ✓ |  |  | 1. Introduction; Clinics in Rehabilitation (CIR), a multi-centre pain rehabilitation clinic in the Netherlands |
| 1.7 | *The target patient population* | ✓ |  |  | 2.1 Patient pilot study group |
| 1.8 | *The intended use of the ML model* | ✓ |  |  | 2. Methods; Combined prognostic patient profile of 13 machine learning models to provide clinical decision support for IMPT clinicians for the predicted success of treatment |
| 1.9 | *Existing model performance benchmarks for this task* | ✓ |  |  | Performance of Framework of 13 machine learning models in the prognostic patient profile reported with initial development project (Zmudzki & Smeets, 2023) |
| 1.10 | *Ethical and other regulatory approvals obtained* | ✓ |  |  | 2.2 Data sources and preprocessing |
| **The Data** | | | | | |
| 2.1 | *Inclusion / exclusion criteria for the patient cohort* | ✓ |  |  | 2.1 Patient pilot study group; comparison of pilot study group to initial IMPT patient dataset provided in section 3. Results |
| 2.2 | *Methods of data collection* | ✓ |  |  | 2.1 Patient pilot study group; prospective clinician assessment of new IMPT patients; Data collection challenges provided in section 2.2 Data sources and preprocessing |
| 2.3 | *Bias introduced due to the method of data collection used* | ✓ |  |  | 2.2 Data sources and preprocessing; and section 4.5 Limitations |
| 2.4 | *Data characteristics* | ✓ |  |  | 2.1 Patient pilot study group; and comparison of pilot study group to initial IMPT patient dataset provided in section 3. Results |
| 2.5 | *Methods of data transformations and preprocessing applied* | ✓ |  |  | 2.2 Data sources and preprocessing |
| 2.6 | *Known quality issues with the data* | ✓ |  |  | 2.2 Data sources and preprocessing; and section 4.5 Limitations |
| 2.7 | *Sample size calculation* |  |  | ✓ | The planned small sample for the pilot study aimed to prospectively test the machine learning prognostic patient profiles with new IMPT patients. 2.2 Data sources and preprocessing; and section 4.5 Limitations |
| 2.8 | *Data Availability* | ✓ |  |  | Data availability statement |
| **Methodology** | | | | | |
| 3.1 | *Strategies for handling missing data* | ✓ |  |  | 2.2 Data sources and preprocessing; and section |
| 3.2 | *Strategies for addressing class imbalance* | ✓ |  |  | Class imbalance strategies reported in initial machine learning development project (Zmudzki & Smeets, 2023); And extended strategy involving outcome measure consolidation to exclude models with high true positive and negative rates; section 2.3 2.3. IMPT prognostic profile outcome consolidation |
| 3.3 | *Strategies for reducing dimensionality of data* | ✓ |  |  | Feature selection for the 13 machine learning models undertaken separately for each outcome, reported previously (Zmudzki & Smeets, 2023) |
| 3.4 | *Strategies for handling outliers* | ✓ |  |  | The original patient dataset for the machine learning development was cleaned and validated to verify outlier errors, the supervised learning included several machine learning models for each outcome to assess best performing algorithms in the case of skewed data or outliers; previously reported (Zmudzki & Smeets, 2023) |
| 3.5 | *Strategies for data augmentation* |  |  | ✓ | No synthetic data were generated for the machine learning training, all models were trained on source patient data, previously reported (Zmudzki & Smeets, 2023) |
| 3.6 | *Strategies for model pre-training* | ✓ |  |  | All machine learning models were trained using random 5 fold cross validation, and tested through withholding random portions of the initial patient data. There has been no pretraining on source data as interim step for further training on the current prognostic pilot study; (Zmudzki & Smeets, 2023) |
| 3.7 | *The rationale for selecting the machine learning algorithm* | ✓ |  |  | Machine learning models were selected on best performance for each of the 13 separate outcome measures, (Zmudzki & Smeets, 2023) |
| 3.8 | *The method of evaluating model performance during training* | ✓ |  |  | Algorithm performance for each of the 13 models were estimated using 5 fold random partitioning and cross validation, (Zmudzki & Smeets, 2023) |
| 3.9 | *The method used for hyperparameter tuning* | ✓ |  |  | The machine learning models were developed using the MATLAB machine learning toolbox which includes multiple methods for hyperparameter tuning including grid search and Bayesian optimization; (Zmudzki & Smeets, 2023) |
| 3.10 | *Model’s output adjustments* |  | ✓ |  | No explicit output threshold adjustments or weighting for ‘cost’ were made during the initial machine learning model development. |
| **Evaluation** | | | | | |
| 4.1 | *Performance metrics used to evaluate the model* | ✓ |  |  | MATLAB machine learning functions including accuracy, precision, recall, F1-score, AUC-ROC, MSE, RMSE and confusion matrix; used to evaluate each model's performance and ability to handle the IMPT imbalanced outcomes; (Zmudzki & Smeets, 2023) |
| 4.2 | *The cost or consequence of errors* |  | ✓ |  | No cost adjustments were undertaken for the initial development, the performance metrics in 4.1 were assessed and reported; (Zmudzki & Smeets, 2023) |
| 4.3 | *The results of internal validation* | ✓ |  |  | Metrics for error estimation of each of the 13 models previously reported (Zmudzki & Smeets, 2023) |
| 4.4 | *The final model hyperparameters* | ✓ |  |  | The final model hyperparameters have been reported for each of the 13 machine learning models in the IMPT prognostic patient profile (Zmudzki & Smeets, 2023) |
| 4.5 | *Model evaluation on an external dataset* | ✓ |  |  | This prognostic pilot study prospectively evaluated the prognostic patient profiles; section 2. Methods and results sections 3.1, 3.2 and 3.3 |
| 4.6 | *Characteristics relevant for detecting data shift and drift* | ✓ |  |  | The pilot study used a prospective group of new IMPT patients as reported in section 3 results and our initial machine learning development (Zmudzki & Smeets, 2023). Section 4.6 Ongoing and future research directions presents planned new IMPT patient data external validation and machine learning model retraining, which will examine and adjust for potential data shift or drift |
| **Explainability and Transparency** | | | | | |
| 5.1 | *The most important features and how they relate to the outcome(s)* | ✓ |  |  | Feature selection was separately undertaken for each machine learning model (Zmudzki & Smeets, 2023); Section 4.2. Data timing and utilization presents weighted feature selection across the complete prognostic profile and how this could help assessment of data collections, for data items that are low across all outcomes in feature selection |
| 5.2 | *Plausibility of model outputs* | ✓ |  |  | All phases of the machine learning development have been undertaken with close IMPT clinician collaboration for outcome framework development and all testing phases. Section 3 Results indicate the results are consistent with clinician assessment of IMPT patients |
| 5.3 | *Interpretation of model's results by an end-user* | ✓ |  |  | The machine learning prognostic patient profile has been developed for clinician and patient IMPT decision support, with outcomes stratified by clinical domain as presented in section 3.4. Revised prognostic patient profile; including the new prognostic profile summary indicators to assist interpretation |

Table 2: IMPT study group and prospective prognostic patient profile pilot study baseline characteristics

| **Baseline category** | | **Initial study (N=2,364)** | | **Pilot study  (N=17)** | |  |
| --- | --- | --- | --- | --- | --- | --- |
|  |  | **%** | **n** | **%** | **n** | *p-*value |
| **Gender** | |  |  |  |  | 0.791 |
|  | Male | 27.5 | 649 | 29.4 | 5 |  |
|  | Female | 72.5 | 1,715 | 70.6 | 12 |  |
| **Age group** | |  |  |  |  | 0.046 |
|  | < 20 years | 1.4 | 33 |  | 0 |  |
|  | 20 to 29 | 15.3 | 361 | 5.9 | 1 |  |
|  | 30 to 39 | 21.7 | 513 |  | 0 |  |
|  | 40 to 49 | 25.8 | 610 | 29.4 | 5 |  |
|  | 50 to 59 | 24.2 | 571 | 52.9 | 9 |  |
|  | 60 and over | 11.7 | 276 | 11.8 | 2 |  |
| **Pain diagnosis** | |  |  |  |  | 0.138 |
|  | WPN 2 chronic pain syndrome | 0.3 | 7 |  | 0 |  |
|  | WPN 3 chronic pain syndrome | 84.6 | 1,997 | 64.7 | 11 |  |
|  | WPN 4 chronic pain syndrome | 14.0 | 331 | 35.3 | 6 |  |
|  | Other pain syndrome | 0.9 | 21 |  | 0 |  |
|  | Psychiatric disease | 0.2 | 4 |  | 0 |  |
| **Number of pain locations** | |  |  |  |  | 0.035 |
|  | 1 | 10.2 | 231 | 17.6 | 3 |  |
|  | 2-5 | 51.0 | 1,159 | 70.6 | 12 |  |
|  | >5 | 38.9 | 885 | 11.8 | 2 |  |
| **Living status** | |  |  |  |  | 0.313 |
|  | Alone | 17.5 | 397 | 29.4 | 5 |  |
|  | With partner | 65.4 | 1,486 | 58.8 | 10 |  |
|  | Living apart together | 5.2 | 119 | 0.0 | 0 |  |
|  | With parent(s) | 6.5 | 148 | 0.0 | 0 |  |
|  | Other | 5.3 | 121 | 11.8 | 2 |  |
| **Highest level of education** | |  |  |  |  | 0.005 |
|  | No | 0.6 | 14 |  | 0 |  |
|  | Primary school | 2.8 | 63 |  | 0 |  |
|  | Pre-vocational secondary | 13.8 | 312 | 23.5 | 4 |  |
|  | Secondary vocational | 44.2 | 1,003 | 17.6 | 3 |  |
|  | Pre-university | 7.1 | 162 | 11.8 | 2 |  |
|  | Pre-university not finished | 6.4 | 146 | 35.3 | 6 |  |
|  | Higher professional | 23.7 | 537 | 11.8 | 2 |  |
|  | Postdoctoral | 1.4 | 31 |  | 0 |  |
| **Paid employment** | |  |  |  |  | 0.999 |
|  | No | 36.1 | 819 | 35.3 | 6 |  |
|  | Yes | 63.9 | 1,450 | 64.7 | 11 |  |
| **Duration of symptoms** | |  |  |  |  | 0.027 |
|  | 0-3 months | 1.1 | 24 | 0.0 | 0 |  |
|  | 3-6 months | 6.3 | 144 | 0.0 | 0 |  |
|  | 6-12 months | 13.1 | 298 | 41.2 | 7 |  |
|  | 1-2 years | 17.0 | 386 | 0.0 | 0 |  |
|  | 2-5 years | 24.7 | 561 | 29.4 | 5 |  |
|  | More than 5 years | 37.8 | 860 | 29.4 | 5 |  |
| **Use of pain medication** | |  |  |  |  | 0.212 |
|  | No | 37.3 | 848 | 52.9 | 9 |  |
|  | Yes | 62.7 | 1,423 | 47.1 | 8 |  |

WPN, classification of the Working Group on Pain Rehabilitation Netherlands (Werkgroep Pijnrevalidatie Nederland)(Nederlandse vereniging van revalidatieartsen, 2020); PDI, pain disability index; PSC, patient-specific complaints; NRS, numeric rating scale; CIS, checklist individual strength; SF-12 PCS, short-form quality of life survey physical component score. Minor variations in subgroup sample sizes were due to missing data. *P* values calculated using Fisher’s exact test.

Consistent with IMPT populations in the Netherlands (Köke et al., 2017) and baseline characteristics of our initial project study group, a high proportion of pilot study patients were women (70.6%). The pilot study patient characteristics (N=17) show a high proportion of female patients (70.6%), consistent with our initial IMPT study group (72.5%, N =2,364), (Table 2). Mean (±SD) patient age was not significantly different from the initial study with patients being 7.6 years older; 51.4 ± 8.6 years versus 43.8 ± 13.0 years. As described in the methods, calculation of *p*-values as a measure of association are not definitive metrics (Hayes-Larson, Kezios, Mooney, & Lovasi, 2019); and there are limitations with using Fisher’s exact test even in small sample sizes (Amiri & Modarres, 2017). For these reasons the *p*-values presented in (Table 2) are provided as indicative to highlight the most prominent associations.

Characteristics that were notably different include; most pilot study patients experienced pain in multiple anatomical locations, with 82.4% reporting more than one (compared with initial study 89.9%) and 11.8% indicating more than five locations (lower than 38.9% in the initial study); more of the pilot group attained a higher level of education; and duration of pain symptoms in the pilot study group report a higher proportion of 6-12 months and a lower proportion with symptoms over 5 years. Although most patients in the initial study data (65.7%) were overweight with a body mass index (BMI) ≥ 25 and 30.4% met the threshold considered obese (BMI ≥ 30), our machine learning models showed that BMI was not a selected prognostic feature in any of the outcome models. For this reason, BMI is not compared for the pilot study group as it was not extracted for prognostic profile development.

## Prognostic patient profile performance

Each IMPT prognostic patient profile was individually prepared prior to initial patient assessment, ready for clinician review and patient discussion. The summary of predicted outcomes across each dimension and outcome measure is provided in (Table 3) for each patient across the 3 rounds of pilot testing. The positive majority indicator is a simple count of positive outcomes of the total 13 profile measures. Positive individual outcomes and positive majority indicators are shown as green and negative outcomes and majority indicators as red shaded cells. For the initial profile review which includes the original full set of 13 outcomes, there are 16 of the 17 patients (94.1%) with a majority positive profile and one patient with a majority negative profile. The table presents the 13 outcome measures group within the five outcome domains of activity / disability, pain, fatigue, coping, and health related quality of life. The figures also present the percentage of patients in the initial patient dataset achieving a positive outcome (column % positive), indicating potential amplified algorithm sensitivity resulting from unbalanced training data. Each outcome measure is also presented with the area under the curve (AUC) statistic as well as true positive (TPR) and true negative rates (TNR). This full set of 13 outcomes was reported in our initial machine learning development research for assessment of outcome measure accuracy (Zmudzki & Smeets, 2023).

Actual clinician assessment of a positive indication for IMPT (or not) is shown at the bottom of (Table 3). The figures show that 13 of the 17 patients (76.5%) were clinician assessed consistently with the prognostic patient profiles. The 5 patients where the prognostic profile did not confirm clinician assessment reflect presentation of the simple majority positive indicator. This majority indicator was a simple summary used to assess the initial study results, with this pilot study planned to assess the performance of the 13 outcomes and consolidation of redundant supplementary measures. Review of the complete 13 outcome results shows that the initial outcomes 2, 4, and 5 all have a true positive rate of 1 and all patients received a positive indication for all 13 measures. The composite summary indicators numbered 8 and 11, as well as the General Perceived Effect (GPE) coping also have true positive rates of or near 1 and were assessed as not contributing materially to the total prognostic profile. For this reason, these 6 outcome measures, which were previously included for algorithm performance review, were removed from the profile. The consolidated prognostic patient profile was therefore reduced from 13 to 7 outcome measures, as presented in the following sections.

Each IMPT prognostic patient profile was prepared before initial patient assessment, ready for clinician review and patient discussion. Table 3 shows patient outcome predictions across the 13 measures, with positive outcomes shaded green and negative outcomes shaded red. Initially, 94.1% of patients had a majority positive profile, but the simple majority indicator was found to be overly sensitive due to unbalanced training data.

Clinician assessments were consistent with prognostic profiles for 76.5% of patients. The initial results for some outcome measures, which had a true positive rate of 1, were deemed redundant and removed. This led to a consolidated profile, reducing the outcome measures from 13 to 7.

The revised 7 outcome framework focuses on the most relevant measures, presenting a more accurate proportion of positive and negative outcomes. This new profile summary indicator categorizes outcomes into positive, negative, and mixed groups, aiding in clearer decision-making for IMPT suitability.

Table 3: Pilot study prognostic patient profiles – complete 13 outcome measures

Source: Machine learning IMPT prognostic patient profile pilot study (N=17). AUC=area under the curve, TPR=true positive rate, TNR=true negative rate. Patient counts provided in (Multimedia Appendix 1).

## Consolidation of outcome measures

The consolidated 7 outcome framework does not reduce prognostic content of the stratified profile but focuses the outcome measures contributing to overall patient assessment. The consolidated prognostic profile results are summarised again in (Table 4). Removal of the high true positive measures shows the revised perspective of the characteristic mix of outcome measures for each patient. The removed outcome measures were misleadingly adding positive outcomes across all patients producing overinflated counts of positive measures, and a corresponding overinflated proportion of majority positive profiles. Where the 13-outcome view in (Table 3) showed 16 of the 17 (94.1%) patients with a positive majority, the consolidated profile reduces the positive majority to 7 of 17 (41.2%). The consolidated profile reduces the number of consistent clinician assessments to 12 of 17 (70.1%), from the previous 13 of 17 (76.5%). This results from the more accurately presented proportion of patients with a characteristic mix of positive and negative predicted outcomes.

This illustrates the limitation of the simple positive majority perspective, and the potential for more useful and accurate summary indicators. Examination of consistent clinician assessment results show the prognostic patient profiles that were not consistent were mixed, but still included at least 1 positive outcome. This provides the perspective that within the high number of positive IMPT outcomes, there is a spectrum from all positive outcome measures (7/7), to most positive, and further to a few or single positive outcome, still potentially sufficient to establish a personal IMPT goal and positive result.

Table 4: Pilot study prognostic patient profiles – consolidated 7 outcome measures
 Source: Machine learning IMPT prognostic patient profile pilot study (N=17). AUC=area under the curve, TPR=true positive rate, TNR=true negative rate. Patient counts provided in (Multimedia Appendix 1).

The motivation for the new summary indicator was to redefine the negative category from a simple majority (4/7 outcomes) to all 7 of 7 measures negative, and positive as 3 or more outcomes positive. Both groups represent positive and negative summary indication with increased confidence. The remaining profiles are now grouped as ‘mixed’. This distinction is useful to articulate as it highlights patients with provisional positive indication subject to further assessment. In this case the prognostic profiles still provide specific outcome dimensions and outcomes to support further assessment and potential discussion with the patient. For this reason, as described in the methods, we developed a new profile summary indicator defined with 3 categories (positive, negative and mixed), as presented in the following section.

## New prognostic profile summary indicators

This section presents the consolidated profile including multiple new summary indicators in the top section of (Table 5). The majority positive indicator is presented as previous for reference, including the count of positive outcomes. The new summary indicator now shows 13 positive, 1 negative and 3 mixed profiles. As these newly defined positive and negative groups are more clearly articulated, 12/14 (85.7%) are consistent with clinician assessment. For the remaining 3 patients with mixed positive and negative outcome measures, the prognostic profile was not used in 2 cases, one where the patient turned out to have no request for help with pain rehabilitation, and another where the planned assessment was rescheduled beyond the pilot study period, shown as profile not used in (Table 5). In 2 of the 3 mixed cases the profiles were predominantly negative, and clinician confirmed as negative during assessment, adding to the total number of 14/17 (82.4%) prognostic patient profiles consistent with clinician assessment.

Clinician assessment notes indicate the stratified prognostic profiles were discussed with around half of the study group (8/17, 47.1%), shown in lower section of (Table 5). The patient review discussion was helpful for confirming profiles that were mostly positive, verifying the positive decision. The review discussions were also reported as helpful in 2 additional cases, one where review of the individual negative outcomes helped the patient decide not to proceed with IMPT, although a summary indicator was positive, and another mixed case with mostly negative outcomes which also helped confirm a decision not to proceed with IMPT. This has shown that the profile was of value to realistically discuss the possibility that IMPT might not provide a positive result, supporting patients to consider whether the uncertainty of success was worth investment of time and effort. This is also considered a positive assessment scenario as the prognostic patient profile may assist with the decision to enter IMPT or not, rather than potentially drop out later if progress was not being achieved. Collectively the prognostic patient profiles were clearly consistent with clinician assessment in 12 cases and reported as additionally useful in 3 further cases where the stratified profile helped clinician and patient decision making for whether to enter IMPT (or not).

Table 5: Pilot study prognostic patient profiles – consolidated 7 outcome measures – new summary indicators


Source: Machine learning IMPT prognostic patient profile pilot study (N=17). AUC=area under the curve, TPR=true positive rate, TNR=true negative rate. P=positive, N=negative, M=mixed; profile accuracy H=high, M=medium, L=low; Tick indicates consistent clinician assessment, discussion with patient, profile was helpful in assessment, or profile was not used. Patient counts provided in (Multimedia Appendix 1)*.*

The new prognostic profile summary indicators also include an initial profile accuracy category for assessment of predictive accuracy across each profile. Although the pilot study is a small sample, the results consistently indicated that all 6 patients with a ‘high’ accuracy category were confirmed to have a clinician consistent prognostic profile. Many of this high accuracy category group (4/6) had a clear positive or negative profile summary, highlighted by the final indicator where all 7 outcomes are consistently positive or negative.

The small pilot test sample (n=17) interestingly provided representative similar distributions of expected majority outcomes. As reported previously using the initial 13 outcome profile, around 92% of patients had positive indicators, reflecting the generally positive outcomes from the program (Zmudzki & Smeets, 2023). The pilot patient study group using the complete 13 outcomes is indicatively consistent with proportions of positive and negative majority positive profiles, (Table 6). The consolidated 7 outcome prognostic profiles similarly correctly identify the negative profile but present the revised split of positive profiles between majority positive with increased confidence, as well as the profiles with mixed positive and negative outcomes.

Table 6: IMPT prognostic patient profile summary indicator of positive, mixed and negative patients

| Patient prognostic profile summary indication | Baseline  (n) | Baseline  (%) | Profile 13 (n) | Profile 13 (%) | Profile 7 (n) | Profile 7 (%) |
| --- | --- | --- | --- | --- | --- | --- |
| Positive | 1,806 | 92.5% | 16 | 94.1% | 13 | 76.5% |
| Mixed | n/a | n/a | n/a | n/a | 3 | 17.6% |
| Negative | 147 | 7.5% | 1 | 5.9% | 1 | 5.9% |
|  | 1,953 | 100.0% | 17 | 100.0% | 17 | 100.0% |

Source: Baseline positive majority results (Zmudzki & Smeets, 2023), n/a= not applicable

# IMPT prognostic variables weighted by minimum redundancy maximum relevance scores.


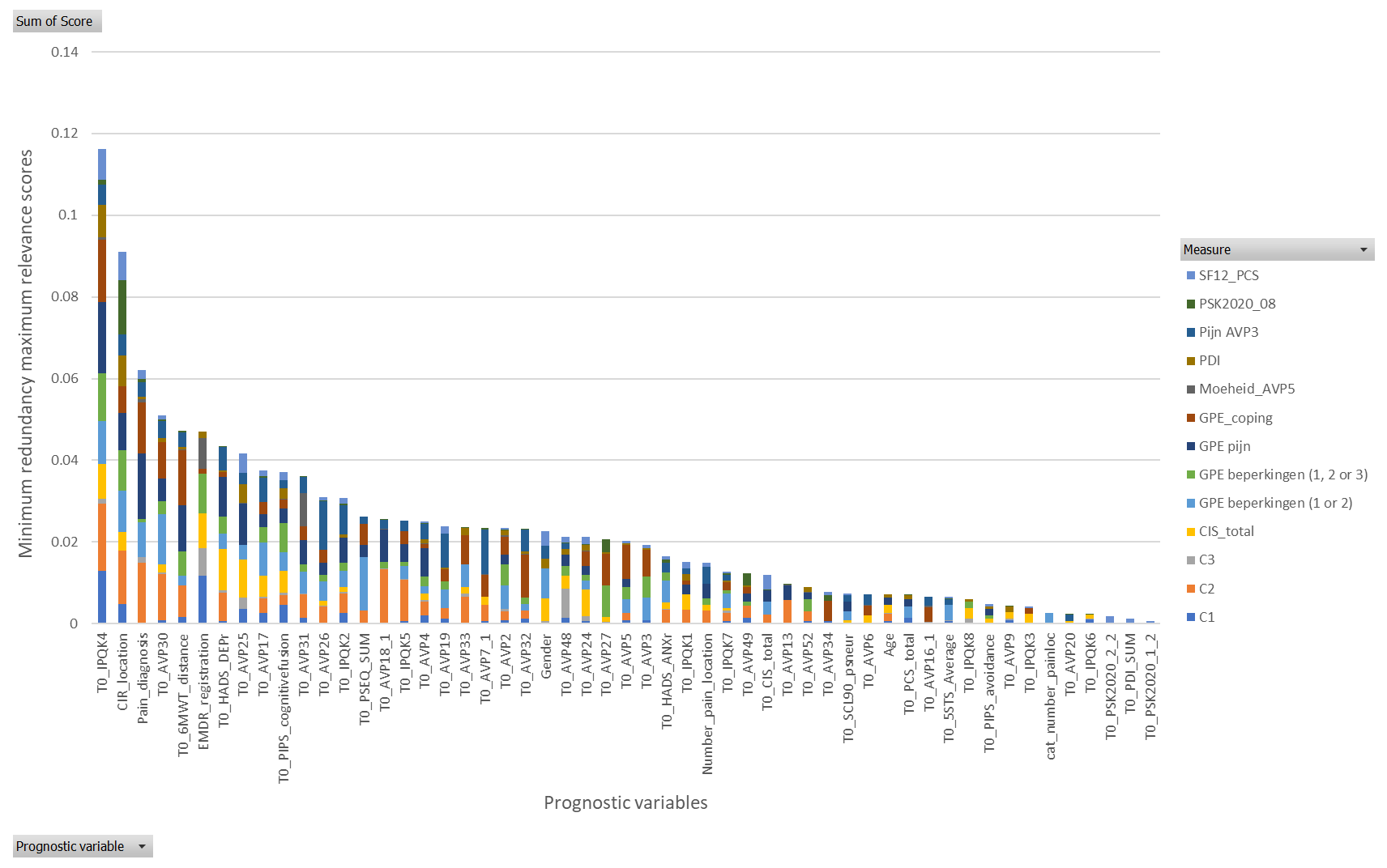

Supplement: Multimedia Appendix 1 [file rehab-v12-e65890-s001.docx]
